# Binocular function to increase visual outcome in patients implanted with a diffractive trifocal intraocular lens

**DOI:** 10.1186/s12886-015-0089-9

**Published:** 2015-08-21

**Authors:** Florian T. A. Kretz, Matthias Müller, Matthias Gerl, Ralf H. Gerl, Gerd U. Auffarth

**Affiliations:** Department of Ophthalmology, International Vision Correction and Research Centre (IVCRC) & David J Apple International Laboratory on Ocular Pathology, University of Heidelberg, Germany, Im Neuenheimer Feld 400, 69120 Heidelberg, Germany; Augenklinik Ahaus_Raesfeld-Rheine, Gerl Group, Ahaus, Germany, Am Schlossgraben 13, 48683 Ahaus, Germany

## Abstract

**Background:**

To evaluate binocular visual outcome for near, intermediate and distance compared to monocular visual outcome at the same distances in patients implanted with a diffractive trifocal intraocular lens (IOL).

**Methods:**

The study comprised of 100 eyes of 50 patients that underwent bilateral refractive lens exchange or cataract surgery with implantation of a multifocal diffractive IOL (AT LISA tri 839MP, Carl Zeiss Meditech, Germany). A complete ophthalmological examination was performed preoperatively and 3 month postoperatively. The main outcome measures were monocular and binocular uncorrected distance (UDVA), corrected distance (CDVA), uncorrected intermediate (UIVA), and uncorrected near visual acuities (UNVA), keratometry, and manifest refraction.

**Results:**

The mean age was 59.28 years ± 9.6 [SD] (range 44–79 years), repectively. There was significant improvement in UDVA, UIVA, UNVA and CDVA. Comparing the monocular results to the binocular results there was a statistical significant better binocular outcome in all distances (UDVA *p* = 0.036; UIVA *p* < 0.0001; UNVA *p* = 0.001). The postoperative manifest refraction was in 86 % of patients within ± 0.50 [D].

**Conclusions:**

The trifocal IOL improved near, intermediate, and distance vision compared to preoperatively. In addition a statistical significant increase for binocular visual function in all distances could be found.

**Trial registration:**

German Clinical Trials Register (DRKS) DRKS00007837

## Background

In todays world patients’ expectation in regards to refractive outcome and spectacle independence have increased substantially and even cataract patients have the same demands as refractive patients.

For the ophthalmologist this means that the final goal is to achieve an accuracy of the target refraction with less than ±0.5 D postoperatively [1].

Recent advances in microsurgery and the latest developments in intraocular lenses (IOLs) have allowed surgeons to achieve more accurate and predictable postoperative refractive results. Still, in order to achieve spectacle independence the simultaneous treatment of presbyopia is crucial.

In the field of lens surgery the use of multifocal IOLs has shown that it can improve uncorrected near visual acuity (UNVA) and uncorrected distance visual acuity (UDVA) and therefore reduce spectacle dependence [2–6]. Toward this purpose, many designs based on different optical principles have been applied in the manufacturing of IOLs. Basically, four types of IOLs are available; refractive, diffractive, refractive–diffractive, and accommodating. Although all can improve UNVA and UDVA, there are collateral effects that should be avoided, such as halos, glare, and loss of contrast sensitivity [3–5, 7]. Regarding uncorrected intermediate visual acuity (UIVA) a great variability in results has been observed with the use of different multifocal IOL models. Therefore, improvement in intermediate vision is still needed to increase the level of patient satisfaction.

**Fig. 1:**
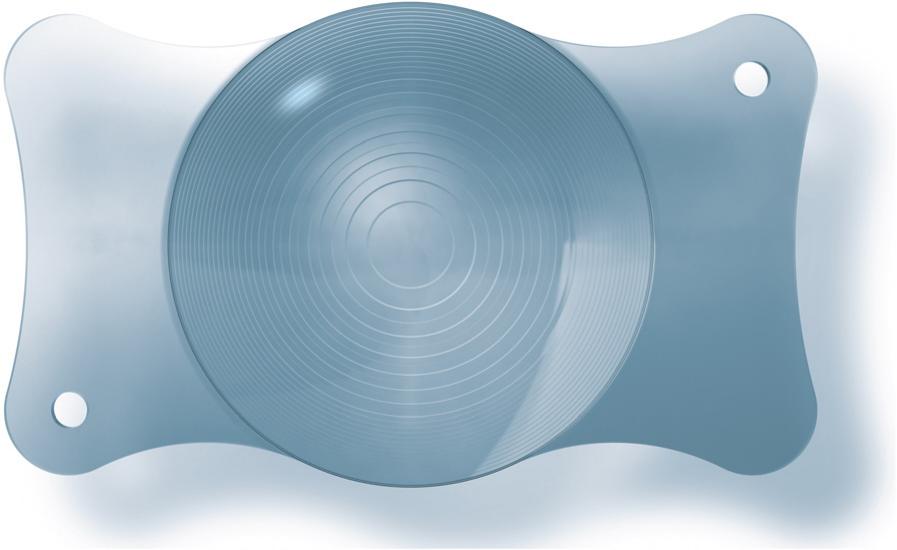
AT LISA tri 839MP (Calr Zeiss MEditech, Jena, Germany)

With a change in society and especially the demands in daily work force (use of computers, tablets, smart phones) excellent intermediate vision is becoming a greater demand of our patients. Newer IOL models, which create a true intermediate focus, have proven to fulfill this needs [8–10]. In the present study, bilateral implantation of the AT Lisa tri 839MP (Carl Zeiss Meditec AG), a new diffractive IOL with a trifocal design, was evaluated. (Figure. 1) To our knowledge, this is the first larger study (50 patients) to compare binocular visual function to monocular visual function in pseudophakic patients implanted with this trifocal MIOL model and one of the few studies of trifocal IOL technology [8–13].

The purpose of this study was to evaluate and to compare the monocular to the binocular visual results obtained for distance, intermediate, and near visual acuity to evaluate the effect of binocular function after implantation of the trifocal IOL. Surgical complications during the follow- up, subjective patients questionnaire for patient satisfaction were also evaluated.

## Methods

### Patients

In this prospective clinical study, 50 patients were enrolled. Included were patients with bilateral cataract or presbyopia/pre-presbyopia suitable for refractive lens exchange and seeking for spectacle independence. Exclusion criteria were a history of glaucoma or retinal detachment, corneal disease, regular corneal astigmatism greater 0.75D, irregular corneal astigmatism, abnormal iris, macular degeneration or retinopathy, neurophthalmic disease, history of ocular inflammation, previous ocular surgery, unilateral amblyopia or stereopsis less than 200 arc sec. In all cases, binocular cataract surgery with implantation of the trifocal IOL AT LISA tri 839MP (Carl Zeiss Meditec) was performed (Fig. 1) The ethics committee of the doctors chamber Westfalen-Lippe and the medical faculty of the Westfälischen Wilhelms-University, Münster approved the study under the reference number: 2014-078-b-S. All patients were adequately informed and signed a consent form. The study adhered to the tenets of the Declaration of Helsinki.

### Clinical protocol

Before surgery, a complete ophthalmological examination was performed, including manifest refraction, keratometry, uncorrected (UDVA) and corrected distance visual acuity (CDVA), Stereopsis (Lang Test II) Goldmann applanation tonometry, slit lamp examination, corneal topography, biometry (IOL Master v.4.3, Carl Zeiss Meditec), and fundoscopy. Postoperatively, patients were examined the day after surgery as well as at 3 month (range:2.5–4 months) after surgery of the second eye. The postoperative examination protocol at 3 months was identical to the preoperative protocol, with the additional evaluation of monocular and binocular uncorrected intermediate visual acuity (UIVA) at 66 cm, monocular and binocular uncorrected near visual acuity (UNVA) at 40 cm. In addition, patients were asked about their satisfaction with the results of the surgery as well as about the perception of photic phenomena, such as glare or halos. Patient satisfaction criteria were spectacle independence for: reading newspaper, reading books, watching TV, driving at day time, driving at night time, shopping at the supermarket, performing computer work, performing house and garden work and performing fine work. The answers consisted of yes, partly, no and not applicable.

As all eyes had an axial length >21.00 mm and < 29.00 mm, the Haigis formula was used for the calculation of the IOL power according to the measurements of corneal power, axial length, and anterior chamber depth obtained with the IOLMaster v4.3 (Carl Zeiss Meditec) system. Target refraction was closest to emmetropia in all cases.

### Intraocular lens

As described in previous studies the AT Lisa tri 839MP (Fig. 1) is a preloaded IOL with a single-piece diffractive multifocal design. It has a 6.0 mm biconvex optic and an overall length of 11.0 mm. It is a foldable hydrophilic acrylate IOL with a water content of 25 % and hydrophobic surface properties [9]. The near add is +3.33D and the intermediate add is +1.66D. The central 4.34 mm follow the described trifocal design, while the peripheral part is only bifocal.

### Surgery

All surgeries were performed using a standard technique of sutureless 2.2 mm phacoemulsification. All incisions were made at the 12 o’clock position. Retrobulbar anaesthesia and mydriatic drops were instilled in all cases prior to the surgical procedure. After capsulorhexis creation with a cystotome under current irrigation through the cystotome needle, phacoemulsification and bimanual cortex peeling with capsule polishing, the IOLs were inserted into the capsular bag using the BLUEMIXS 180 injector (Carl Zeiss Meditec) through the main incisions. At the end of the surgery subconjunctival injection of dexamethasone and cefuroxime was administered. A postoperative therapy based on a combination of topical antibiotic and steroid was prescribed to be applied four times daily during two week.

### Statistical analysis

SPSS statistics software package version 15.0.1 for Windows (IBM, Armonk, NY, USA) was used for statistical analysis. The Kolmogorov-Smirnov test was used to check the normality of the data distribution. When parametric analysis was possible, the Student *t* test for paired data was performed for all parameter comparisons between preoperative and postoperative examinations as well as between consecutive postoperative visits. Otherwise, when parametric analysis was not possible, the Mann–Whitney test was applied to assess the significance of differences between consecutive examinations. In all cases, the same level of significance (*p* < 0.05) was considered.

## Results

The study enrolled 100 eyes of 50 patients. 42 % were male and 58 % were female. The median patient age was 59.28 years (±7.6 (SD) (range 44.4–79.2 years). In 36 % a regular cataract surgery was performed, while in 64 % a clear lens exchange for presbyopia correction was performed.

### Preoperative measurements

Table 1 shows the preoperative IOL-Master results. There were no statistical significant differences between the flattest meridian (K1) and the steepest meridian (K2) preoperatively (Mann–Whitney-Test). Regarding preoperative refraction there was no statistical significant difference between sphere (*p* = 0.442), cylinder (*p* = 0.928) or spherical equivalent (*p* = 0.418. All patients had a tested stereopsis of 200 arc sec (Lang Test II).

**Table 1:** Preoperative IOL-Master values (axial length = AL; flattest meridian = K1; steepest meridian = K2; anterior chamber deepth = ACD; interquartiles distance = IQA; right eye = OD; left eye OS)

		Mean	Range	Median	IQA	p-value (Mann–Whitney-Test)
AL [cm]	OD	23.933	21.600–28.590	23.795	1.155	0.962
	OS	23.907	21.430–28.190	23.665	1.328	
	total	23.92	21.43–28.59	23.75	1.293	---
K1 [mm]	OD	7.908	7.300–9.110	7.900	0.267	0.825
	OS	7.916	7.320–8.990	7.925	0.245	
	total	7.912	7.300–9.110	7.92	0.27	---
K2 [mm]	OD	7.747	7.120–8.940	7.720	0.28	0.931
	OS	7.746	7.200–8.630	7.746	0.318	
	total	7.747	7.120–8.940	7.73	0.313	---
ACD [mm]	OD	3.213	2.280–3.750	3.300	0.503	0.502
	OS	3.260	2.280–3.950	3.330	0.48	
	total	3.236	2.280–3.950	3.3	0.49	---

### Visual and refractive outcomes

**Fig. 2:**
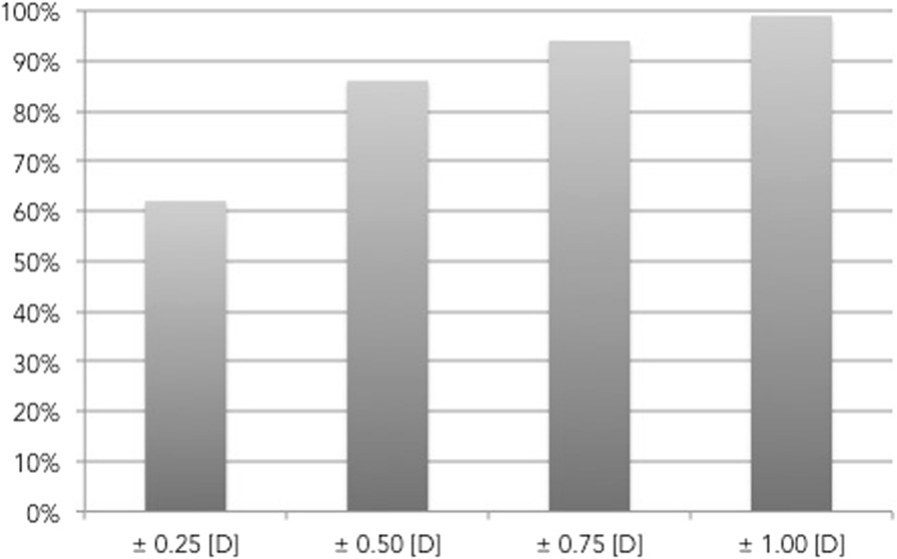
Percentage of patients in deviation of target refraction (Spherical Equivalent [SE])

Table 2 shows the refractive results in all patients over time. There were significant reductions in sphere, cylinder, and spherical equivalent (SE). Postoperatively, the spherical equivalent decreased significantly to 0.08 [D] (*P <* .001) In all patients (table 2). Three months after surgery, the SE ranged from-1.38–0.75 [D] with 86 % of patients being ±0.50 [D] (Fig. 2). In the comparison between OD and OS there was no statistical significant difference between the refractive outcome respectively (*p* = 0.857).

**Table 2:** Refractive and visual results (interquartiles distance = IQA; uncorrected distance visual acuity = UDVA; corrected distance visual acuity = CDVA; uncorrected near visual acuity = UNVA; uncorrected intermediate visual acuity = UIVA)

Mean (Range)	Pre-operative		Post-operative		p-value (Mann–Whitney-Test)
Median (IQA)					
UDVA [LogMAR]	0.72	0.10 to 2.00	0.06	−0.10 to 0.30	<.001
	0.60	0.70	0.10	0.10	
Sphere (D)	−0.05	−13.25 to 6.25	0.20	−0.75 to 1.00	<.001
	1.25	2.25	0.25	0.5	
Cylinder (D)	−0.89	−4.00 to 0	−0.58	−1.75 to 0.00	<.001
	−0.75	0.5	−0.50	0.31	
Spherical equivalent (D)	−0.44	−14.00 to 5.88	−0.08	−1.38 to 0.75	<.001
	0.81	2.66	0.00	0.53	
CDVA [LogMAR]	0.16	0.00 to 1.30	0.04	−0.20 to 0.30	<.001
	0.10	0.3	0.00	0.1	
UNVA [LogMAR]	---		0.06	−0.10 to 0.30	---
			0.05	0.10	
UIVA [LogMAR]	---		0.09	−0.10 to 0.30	---
			0.10	0.10	
binocular UDVA [LogMAR]	---		0.04	−0.10 to 0.20	---
			0.00	0.10	
binocular UNVA [LogMAR]	---		0.01	−0.10 to 0.20	---
			0.00	0.19	
binocular UIVA [LogMAR]	---		0.04	−0.10 to 0.20	---
			0.00	0.10

**Fig. 3:**
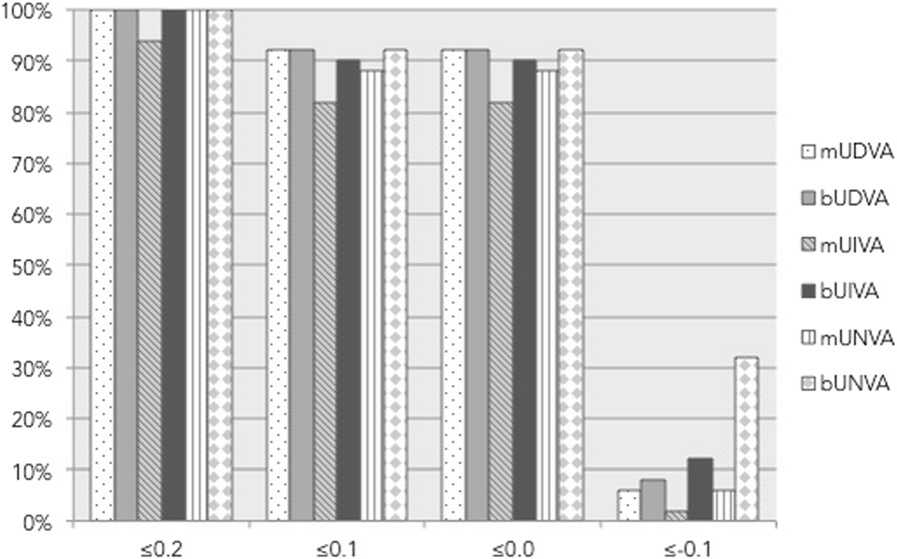
Cumulative uncorrected visual acuity [logMAR] in distance intermediate and near (*n* = 50) mUDVA – monocular UDVA, bUDVA – bincoluar UDVA, mUIVA – monocular UIVA, bUIVA – binocular UIVA, mUNVA – monocular UNVA, bUNVA – binocular UNVA

All patients achieved a postoperative stereopsis equal to the preoperative value of 200 arc sec. Table 2 shows the visual acuities. There was a statistically significant improvement between preoperatively and 3 months postoperatively in the following variables: UDVA, CDVA. By comparing monocular to binocular UDVA, UIVA and UNVA after surgery, there was a statistical significant increase in all distances (Table 3). Figure 3 shows the cumulative visual acuity for the better eye and binocular. 91 % of patients reached binocular UDVA of 0.0 logMAR, 79 % binocular UIVA of 0.0 logMAR and 87 % binocular UNVA of 0.0 logMAR. 100 % of patients reached at last binocular UDVA, UIVA and UNVA of 0.3 logMAR or better (Fig. 3). The effect of binocular fusion gave an average gain of one line for distance, intermediate and near visual acuity. There were no statistical significant differences between the postoperative monocular and binocular UDVA, UIVA, UNVA or the binocular visual acuity gain between the cataract and the refractive lens exchange patients.

**Table 3:** Comparision of monocular and binocular uncorrected visual acuity 3 month postoperatively (interquartiles distance = IQA; uncorrected distance visual acuity = UDVA; uncorrected near visual acuity = UNVA; uncorrrected intermediate visual acuity = UIVA)

Mean (Range)	Monocular		Binocular		p-value (Mann–Whitney-Test)
Median (IQA)					
UDVA [LogMAR]	0.06	−0.10 to 0.30	0.04	−0.10 to 0.20	0.036
	0.10	0.10	0.00	0.10	
UNVA [LogMAR]	0.06	−0.10 to 0.30	0.01	−0.10 to 0.20	<.001
	0.05	0.10	0.00	0.19	
UIVA [LogMAR]	0.09	−0.10 to 0.30	0.04	−0.10 to 0.20	<.001
	0.10	0.10	0.00	0.10

### Patient satisfaction

Seventy prercent of the patients were able to read newspaper and book print without glasses, while the other 30 % answered the question with a partly use of spectacles. All patients were able to watch TV, drive during daytime, go shopping at the supermarket and perform house and garden work without the use of glasses. For nighttime driving 90 % did not need glasses anymore while 10 % were still dependent on the use of spectacles. Computer work was graded with 60 % of patients being spectacle free, 20 % being partly spectacle free and the other 20 % answered with not applicable. Performing fine work 50 % were spectacle free, 40 % were partly spectacle free and 10 % answered with not applicable.

### Complications

No serious complications, such as posterior capsule rupture, endophthalmitis, or corneal decompensation, occurred during the follow-up.

## Discussion

Multifocal IOLs were designed to improve vision at different distances by increasing the depth of field in the eye [14]. The approach is different depending on the particular IOL model; however, the principal goal is to provide the best levels of spectacle independence [2]. As the intermediate focus has become more important for daily life activities, new optical designs try to fulfill those needs. Developments for optical designs have been made for refractive, diffractive, or combinations of booth. One approach is to reduce the add power of MIOLs to shift the focal point closer to an intermediate direction, resulting in a loss of spectacle independence in the near. The monovision approach in those cases increases spectacle independence but reduces stereopsis in near and intermediate distance like known from monofocal IOLs [15]. More recently, accommodating IOL models are being tested and new technologies are being developed. Another technical approach to define true intermediate vision has been the development of trifocal IOLs [8, 10–12, 16, 17].

This third focal point that is induced by a second diffractive structure, also reduces the loss of light compared to other diffractive, bifocal IOLs, by offering true intermediate visual acuity [18]. By giving patients the focal points for both eyes, stereopsis should be obtained.

To our knowledge, this is the first study to compare the results of binocular visual acuity to the monocular visual acuity in a study cohort of 100 eyes of 50 patients having been implanted with a AT LISA tri 839MP in both eyes.

Our UDVA results were notable, with a mean value of 0.06 monocular, with still a statistical significant increase to 0.04 binocular (Table 3). We could also show a similar, statistical significant effect of binocular fusion for UNVA and UIVA (Table 3). We believe these values monocular and especially binocular are sufficient to obtain a high level of spectacle independence for daily life routine.

In the present study, there was an improvement in UDVA and CDVA compared to preoperatively (Table 2). Therefore, the trifocal IOL we tested was very effective. These results are consistent with the refractive values obtained. All were within the interval of -0.50 to +0.50 [D] of SE 3 months after surgery. In a recent study of another trifocal model (Finevision, PhysIOL S,A), Sheppard et al. [12] reported a mean monocular UDVA and CDVA of 0.19 ± 0.09 and 0.08 ± 0.08, respectively. These results are consistent with those presented by Voskresenskaya et al. [10] for the MIOL-Record 3 model (Reper). Our results for both variables (mean 0.06 ± 0.08 for UDVA and 0.04 ± 0.08 for CDVA) seem to be very similar to those published by Lesieur [11] and for the Finevision IOL (mean 0.00 ± for UDVA and 0.00 ± 0.00 for CDVA).

Often described, but never proven is the fact that binocular visual function in MIOL patients leads to an increase of visual acuity in all distances. Our results clearly show a statistical significant increase of UDVA, UIVA and UNVA by comparing monocular to binocular results. Similar results of increased binocular visual acuity compared to monocular visual acuity have been shown in the past. Cagenello R et al. [19] found an average increase of 11 % in a study of four phakic young patients. By recalculating our gain in a percentage, we found a similar binocular summation of around 10 % with an average age of 59 years compared to the 11 % of Cagenello et al. [19] with four patients in an age range of 29–40 years old. Rubin et al. [20] showed in a population based study of 2520 patients in the age between 65 and 84 years an average gain by binocular summation of 1.5 ETDRS letters which was statistical significant, but not clinical significant. As this was a population based study, there were no differentiations between phakic, cataractouse or pseudophakic eyes which limits the possible comparison to our study with a statistical significant binocular gain of 1 line, which we do consider clinical relevant as well. Pardhan S. [21] showed in their study a functional gain of 31-32 % in cataract patients, but no comparison in pseudophakic patients. To our knowledge, our data is the first to compare a larger study group of 50 pseudophakic patients implanted with the same trifocal MIOL showing similar results compared to other studies of mainly phakic patients in various age ranges [19–21].

All patients reported that the final result, as a whole, was excellent or very good. Moreover, all reported that they were comfortable performing intermediate-distance tasks.

The main advantage of multifocal IOLs is the capability of generating different foci to achieve acceptable levels of vision at far and near [16]. In addition, others have shown the advantages of multifocal IOLs over monofocal IOLs [22]. However, better intermediate vision results are still necessary. As the binocular summation adds a statistical increase of visual acuity in all distance, it is favorable to implant the same IOL model with a similar target refraction in both eyes to have an extra gain of visual acuity

## Conclusion

In conclusion, we believe that the excellent visual results obtained at different distances with the trifocal design indicate an emerging technique in the field of the diffractive IOLs. Binocular summation has an impact on clinical and statistical significant increase of visual acuity in all distances to provide a fully functional vision including functional stereopsis for our patients. By trying to find the best possible outcome for cataract and especially refractive lens exchange patients the latest version of trifocal, multifocal IOLs seem to be a favorable option to create spectacle independence in various distances.
